# Cell therapy: A potential solution for the healing of bone cavities

**DOI:** 10.1016/j.heliyon.2020.e05885

**Published:** 2021-01-05

**Authors:** Sara El-Gindy, Maram Farouk Obeid, Kareim Mostafa Elbatouty, Elham Elshaboury, Ehab Hassanien

**Affiliations:** a Department of Endodontic, Faculty of Dentistry, Egyptian Russian University Cairo, Egypt; b Department of Endodontic, Faculty of Dentistry, Ain Shams University Cairo, Organization of African Unity St, El-Qobba Bridge, Al Waili, Cairo Governorate, Egypt; c Department of Endodontic, Faculty of Dentistry, Modern Science and Arts – MSA, Egypt

**Keywords:** Bone healing, Bone marrow mononuclear cells, Cell therapy

## Abstract

**Aim:**

To Explore whether the use of autologous BMMNCs as a cell therapy technique will improve the healing of bone cavities in vivo.

**Methodology:**

After achieving proper anesthesia, mononuclear cells were isolated from iliac crest's bone marrow aspirates (BMMNCs). Then access cavity, root canal preparation, and filling were done in third and fourth premolars, followed by amalgam coronal restoration. After that, a flap was reflected and a standardized bone cavity was drilled, the related root-ends were resected and retrocavity was drilled and filled with MTA. Before repositioning the flap, the bone cavity was filled with the desired filling material according to its corresponding group (n = 8): CollaCote group; where collagen scaffold was used, MNC group; in which CollaCote® loaded with isolated BMMNCs were applied, Biogen group; in which BIO-GEN® graft material was applied and finally Control group; where the bone cavities were left empty to heal spontaneously. Evaluations of healing of the bone cavities were done radiographically and histologically.

**Results:**

The MNC group induced the best healing potential with statistical significant difference from other groups.

**Conclusion:**

cell therapy utilizing autologous BMMNCs looks to beat the conventional therapies and convey a significant improvement in the healing of the bone cavity in vivo.

## 1 Introduction

After root canal treatment, failure of healing of preexisting periapical lesion or the development of a new one is considered as an undesired, unsuccessful outcome that requires retreatment [1]^.^ Retreatments are either performed as nonsurgical endodontic retreatment (NSER) or surgical approach which is addressed when NSER is difficult or challenging [2, 3].

Surgical approaches incorporate debridement of apical lesions together with a reshaping of the surrounding bone, the establishment of a proper seal between root canal system and the periradicular tissues [4]. Unfortunately, following endodontic surgery, periapical bone healing can be directed toward repair or regeneration, depending on various issues, such as the size of the lesion, the availability of cells from the host and biological factors possibly stimulating the healing process in this area [5]. This has directed researchers to focus on different methods to promote adequate healing by bone regeneration rather than fibrous repair. One of these methods is the usage of bone-replacing biomaterials (bone grafts) [6, 7] as an adjunct to endodontic surgery.Animal-derivative preparations are one type of bone graft that has a structure like the human bone and can accelerate the osteoconduction process. BIO-GEN® (Bioteck, Italy) is an excellent example that contains biological apatite of bones, with fewer hydroxyl groups and a larger number of carbonic ions [7].

The second method addressed to achieve bone regeneration is the use of resorbable and non-resorbable barrier membranes [7]. CollaCote® (Zimmer Dental, Carlsbad, CA, USA) is soft, white, pliable, porous, non-friable absorbable collagen wound dressings used in dental surgery. The collagen content of this scaffold allows easy placement of cells and growth factors with total replacement by natural tissues after degradation [8].

The use of cells (cell therapy approach) is the third way to achieve bone regeneration [9]. Cell therapy refers to cellular material with biological activities that cause the desired effect either in vitro or in vivo [10]. Jäger M et al [11] applied that to enhance allograft bone healing through mesenchymal stem cells implantation with concentrated bone marrow.

Experimental and clinical studies have shown that mononuclear cells frequently obtained from iliac crest bone marrow aspirate (BMMNCs) might be used as an alternative to cultured cells with promising results [12]^.^ Jager M et al. [13] considered them as a novel strategy for bone defect treatment.

To the best of our knowledge, few studies addressed the effect of cell therapy in bone formation, thus our study aimed to evaluate the effect of using BMMNCs as a cell therapy technique on the healing process of bone cavities in dogs.

## 2 Materials and methods

This animal experiment was performed following protocols approved by the Ethical Committee, of the first author organization (FDASU-REC D 041308). 16 pathogen-free male 2-years-old mongrel dogs receiving a regular diet and water were included in our study.

### 2.1 Study design and randomization

The dogs were randomly assigned into 4 equal groups, 4 dogs in each group with a total number of 32 standardized bone cavities (15 mm × 10 mm x 10 mm) created in the area of mandibular third and fourth premolars (2 cavities/dog).

### 2.2 Isolation of BMMNCs

The animal was sedated with intravenous injection of 1 mg/kg ketamine (Amoun Pharmaceutical Co, El-Obour City, Egypt) then general anesthesia was induced by using Ketamine HCl (Keiran; EIMC pharmaceuticals Co., Cairo, Egypt) injected intravenously using a cannula in the cephalic vein at a dose of 5 mg/kg body weight. The anesthesia was maintained by using Thiopental Sodium at a dose of 25 mg/kg body weight. 10 ml Bone marrow (BM) was aspirated from the Iliac Crest [14] then mononuclear cells were isolated. Briefly, bone marrow aspirates were diluted with phosphate-buffered saline (1:3) then carefully layered onto Ficoll (1.077 g/cm^3^, Biochrom, Berlin, Germany), density gradient centrifugation without brake at 800 g for 20 min at room temperature were done. The upper layer containing plasma and platelets was drawn off using a sterile pipette leaving the mononuclear cell layer undisturbed at the interface, the later was transferred to a sterile centrifuge tube with the aid of a sterile pipette and balanced salt solution was added. Centrifugation was repeated at 400 g for 10 min at room temperature then all supernatant was removed to get cell pellet [15]. The pellet was washed twice with 3 mL phosphate-buffered saline then loaded on the collagen scaffold, that was previously cut with sterile scissors to match the size of the bone cavity, with the aid of micropipette to be applied immediately within the bone cavity. Before application, cell counting, and viability was evaluated by the trypan blue exclusion assay [16].

### 2.3 Root canal instrumentation and filling

Teeth isolation was performed using sterilized cotton rolls, and high-speed evacuation was used to control saliva. The teeth were cleaned with 0.12% chlorhexidine gluconate (Listermix; Sigma Pharmaceutical Industries, Quesna, Egypt). After gaining occlusal access to the pulp chambers of the involved teeth, the pulps were extirpated and the root canals were cleaned and shaped using Flex-o-Files (Dentsply Tulsa Dental, Tulsa, OK, USA). The canals were filled with warm vertical compaction of gutta-percha and AH Plus root canal sealer (Dentsply Tulsa Dental). The coronal access cavities in all teeth were then restored with amalgam (Ivoclar Vivadent, Amherst, NY, USA) [17].

### 2.4 Bone cavity preparation

**Figure 1:**
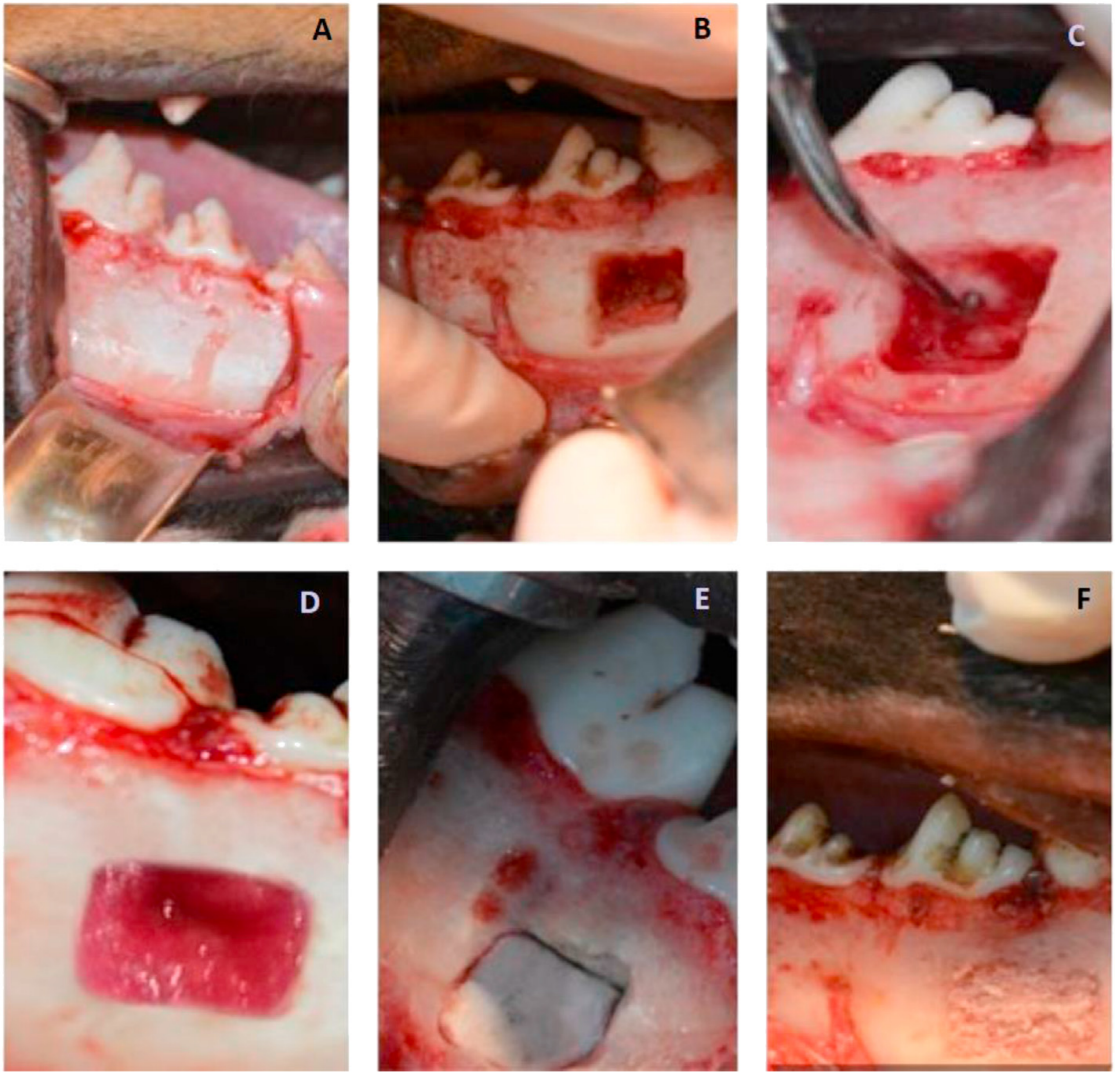
Photographs showing: (A) reflected flab with incisions mesial to the third and distal to the forth premolars, (B) drilled bone cavity, (C) resected root end with retrograde cavity prepared, (D) bone cavity filled with BMMNCs loaded on CollaCote, (E) bone cavity filled with precut CollaCote, (F) bone cavity filled with Biogen.

The first periapical surgery procedure on either the mandibular right or left side was done. Full- thickness mucoperiosteal buccal flap with two releasing incisions (mesial of the third and distal of the fourth premolars) reflected (Figure 1A). The cortical bone covering the root-ends removed using number 6 round bur in a high-speed handpiece and copious saline irrigation then cavity size was adjusted to be (15 mm length x 10 mm width x 10 mm depth)) (Figure 1B). After that, related root-ends (third and fourth mandibular premolars) were resected with a fissure bur in a high-speed handpiece with saline irrigation approximately 3 mm from the apex at an angle approximately 60° to the long axis of the root. Root-end cavities were then prepared to a depth of 3 mm with diamond-coated retrotips (E32D tip, NSK, Satelec, France) (Figure 1C). Root-end cavities were filled with MTA (ProRoot MTA, Dentsply Tulsa, Tulsa, OK) mixed with sterile saline (3:1). Before repositioning the flap, the bone cavities were filled with the desired filling material.

### 2.5 Groups classification

Our groups were named according to the material used to fill the bone cavity (n = 8): **CollaCote group** where collagen scaffold (CollaCote®, Zimmer Dental, Carlsbad, CA, USA) used (Figure 1 E), **MNC group** in which CollaCote® loaded with isolated BMMNCs applied (Figure 1 D) **Biogen group** in which BIO- GEN® (Bioteck, Italy) graft material applied (Figure 1F) and finally **Control group** where the bone cavities left empty to heal spontaneously.

After that, the mucoperiosteal flaps were repositioned and sutured with 4-0 silk sutures. All animals were administered 0.01 mg kg/1 buprenorphine (Buprenex, Reckitt and Coleman Pharmaceuticals, Richmond, VA, USA) subcutaneously for pain control and 300,000 units of penicillin (Bicillin C-R, Wyeth-Ayerst Laboratories, Philadelphia, PA, USA) to prevent infection. After surgery, the animals were placed on a soft diet. Two weeks after completion of the first periapical surgery, the second surgery on the other side was performed following the same protocol. The sutures were removed 2 weeks after each surgery.

### 2.6 Cone-beam computed tomography scanning

After 12 weeks, the dogs were sacrificed using an anesthetic overdose (10 G ketamine Hcl). Jaw segments containing the teeth were removed with the adjacent bone. After fixation with 10% formalin solution, cone-beam radiography was performed using Next Generation I-CAT scanner (Imaging Sciences International, Inc., Hatfield, USA) at 250 kV and 100 mA. Healing of the bone cavity was evaluated quantitively on CBCT scan [18] by measuring the surface area and the volume of the residual bone defects on tomographic sections [19], using Mimics software (version 15.1, Materialize, Belgium). These are inversely proportional to the healing effect, the larger the surface area or volume the worse is the healing.

### 2.7 Histopathological evaluation

1 Decalcification of Jaw segments started using 17% EDTA solution for 120 days. The specimens were embedded in paraffin and then sectioned buccolingually to an average thickness of 6 micro-meter, the sections were stained to be evaluated under a light microscope with Hematoxylin and Eosin stain for assessment of inflammatory cell count [20]. For each slide, three representative fields were analyzed at 8900 magnification. Fields were selected at random from areas having well-preserved tissue with good architecture and intense inflammatory cells. The total inflammatory cell number was counted using Image-J software. The color-coding threshold was calibrated to select the perimeter of the whole inflammatory cells. Then, binary thresholds of the selected color-coded inflammatory cells were finished before calculation. The total number of cells was then counted.
2 Goldner Tri-chrome stain for assessment of the percentage of newly formed bone within the cavity [21]. A rectangular field within the section measuring 1700 × 2200 μm was randomly selected. In this field, the area occupied by bony trabeculae was calculated using ImagePro Plus® software. The percentage of the entire field occupied by bone was then calculated.

### 2.8 Statistical analysis

Cone-beam computed tomographic and histologic images were examined 3 times randomly by 2 observers blinded to the groups at a 1-week interval, each time without the knowledge of the previous results. Weighted coefficient kappa (Kw) was used to measure interobserver reproducibility between observers separately for each time and to measure intra-observer reproducibility between time separately for each observer. Numerical data were explored for normality by checking the data distribution, calculating the mean and median values and using Kolmogorov-Smirnov and Shapiro-Wilk tests. Data showed parametric distribution and were presented as mean, standard deviation (SD) values. One-way ANOVA followed by Tukey's post hoc test was used for statistical analysis. The significance level was set at P < 0.05 within all tests. Statistical analysis was performed with IBM® SPSS® Statistics Version 26 for Windows.

## 3 Results

### 3.1 Isolation of BMMNCS

The aspirated marrow yielded approximately 1X10^6^ Mononuclear cells in the form of a cell pellet.

### 3.2 Radiographic analysis

**Figure 2:**
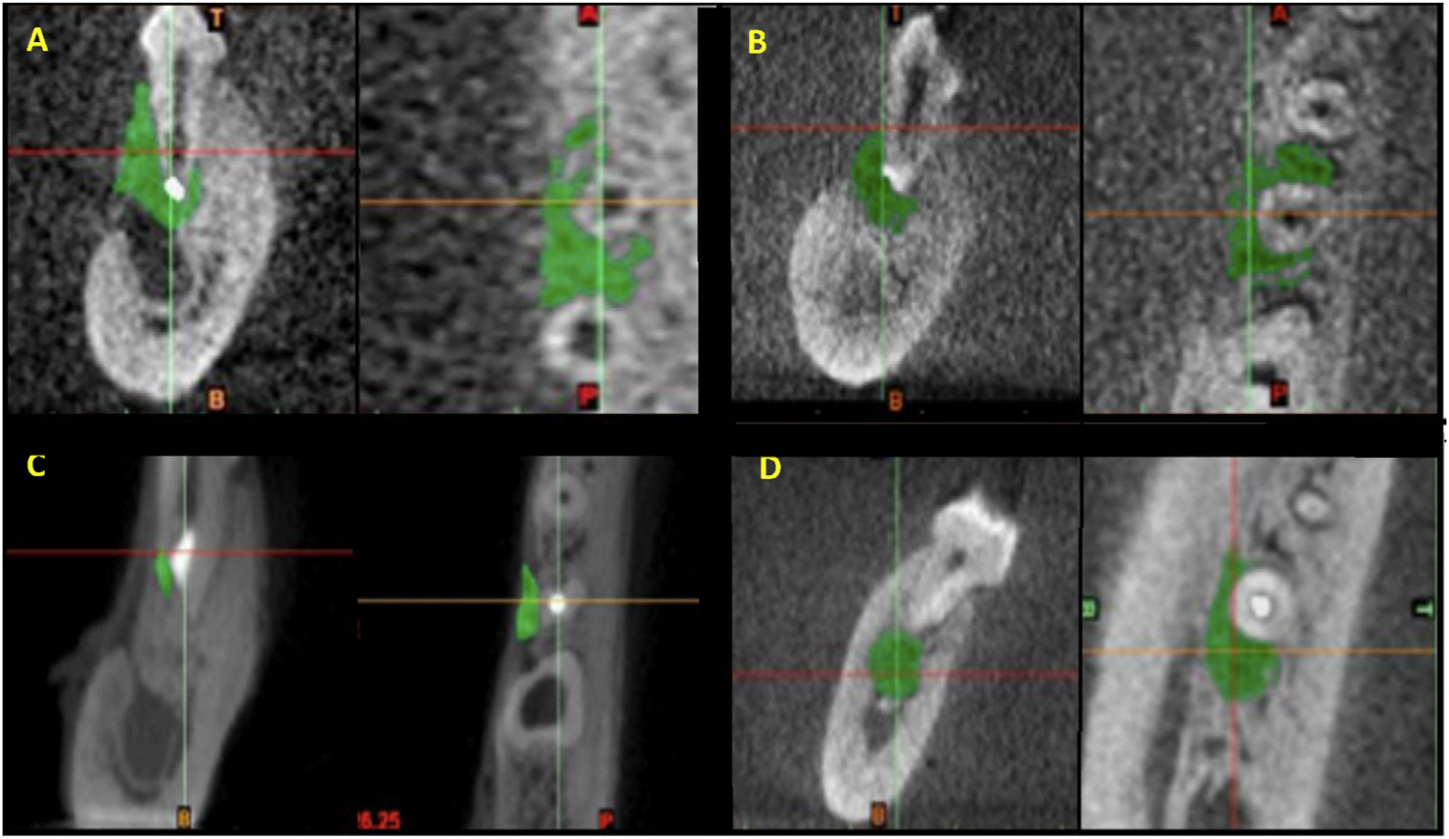
Images of tomographic sections showing the residual bone defect as green colored areas in different groups: (A) Control, (B) Collacote, (C) MNC, (D) Biogen.

The surface area and the volume of the residual bone defects were measured on tomographic sections (Figure 2).

a. Surface area measurement

The mean and standard deviation values are presented in Table 1. There was a statistically significant difference between values measured in different groups (P < 0.001). The highest value was found in the control group (45.8 ± 6.8), followed by Collacote group (37.5 ± 4.5), then Biogen (28.7 ± 4.6) while the lowest value was found with MNC (22.4 ± 4.7). Pairwise comparisons utilizing showed the control group to have a significantly higher value than Collacote group (P = 0.018), Biogen (P < 0.001) and MNC (P < 0.001). In addition, they showed Collacote group to have a significantly higher value than Biogen (P = 0.011) and MNC (P < 0.001) groups. They also showed that there was no significant difference between Biogen and MNC groups (P = 0.100).

b. Volume measurement

**Table 1:** The means, SD values of the surface area in mm^2^ of the residual bone defects in all groups.

Group	Mean	SD	Median	Minimum	Maximum	95% CI		*P*-value
						Lower bound	Upper bound	
Control	45.8^A^	6.8	47.3	34.2	53.8	40.1	51.5	**<0.001∗**
Collacote	37.5^B^	4.5	38.9	30.3	42.7	33.8	41.2	
Biogen	28.7^C^	4.6	28.9	22.1	36.2	24.8	32.5	
MNC	22.4^C^	4.7	22.0	14.5	28.6	18.4	26.3	

∗: Significant at P ≤ 0.05, Means with different superscript letters within the same vertical column are significantly different from each other.

The mean and standard deviation values are presented in Table 2. There was a statistically significant difference between values measured in different groups (P < 0.001). The highest value was found in the control group (117.8 ± 12.8), followed by Collacote group (92.4 ± 4.2), then Biogen (86.4 ± 10.7) while the lowest value was found with MNC (77.0 ± 5.9). Pairwise comparisons showed the control group to have a significantly higher value than Collacote (P < 0.001), Biogen (P < 0.001) and MNC (P < 0.001) groups. In addition, they showed Collacote group to have a significantly higher value than MNC (P < 0.001). They also showed that there was no significant difference between (Collacote and Biogen) (P = 0.560) and between (Biogen and MNC) (P = 0.192).

**Table 2:** The means, SD values of volume in mm^3^of the residual bone defects in all groups.

Group	Mean	SD	Median	Minimum	Maximum	95% CI		*P*-value
						Lower bound	Upper bound	
Control	117.8^A^	12.8	119.5	98.0	132.0	107.0	128.5	**<0.001∗**
Collacote	92.4^B^	4.2	91.0	88.0	100.0	88.8	95.9	
Biogen	86.4^BC^	10.7	87.0	74.0	105.0	77.5	95.3	
MNC	77.0^C^	5.9	78.0	68.0	86.0	72.0	82.0	

∗: Significant at P ≤ 0.05, Means with different superscript letters within the same vertical column are significantly different from each other.

### 3.3 Histologic analysis

a. Inflammatory cell count

**Figure 3:**
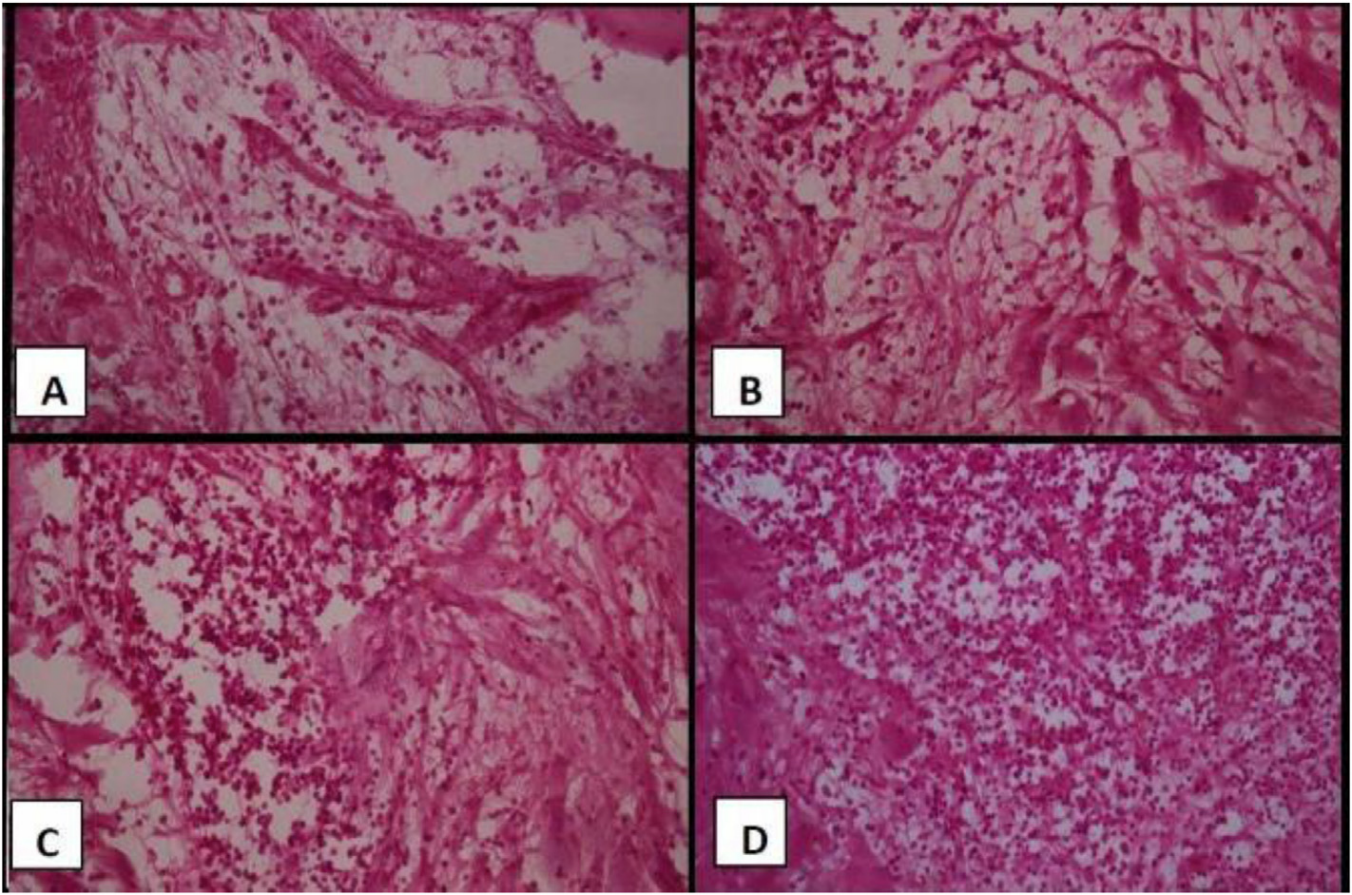
Light microscope photo with H&E stain showing (A) MNC: scattered organized bundles of collagen with inflammatory cells throughout the specimen, (B) Biogen: lesser organized fibrils with noticeable inflammatory cells infiltration, (C) Collacote: more organization and aggregation of collagen fibers with obviously seen inflammatory cells infiltration, (D) Control: marked infiltration of inflammatory cells with scattered and non-organized collagen fibers. (X400).

The mean and standard deviation values are presented in Table 3. There was a statistically significant difference between values measured in different groups (P < 0.001). The highest value was found in the control group (575.6 ± 31.8), followed by Collacote (388.1 ± 55.9), then Biogen (284.2 ± 21.3) while the lowest value was found with MNC (199.7 ± 13.6). Pairwise comparisons showed all groups to have a significantly different values from each other (P < 0.001) (Figure 3).

b. Percentage of new bone:

**Table 3:** The means, SD values of inflammatory cells count in all groups.

Group	Mean	SD	Median	Minimum	Maximum	95% CI		*P*-value
						Lower bound	Upper bound	
Control	575.6^A^	31.8	580.0	524.0	610.0	536.1	615.1	**<0.001∗**
Collacote	388.1^B^	55.9	371.5	320.0	456.0	318.8	457.4	
Biogen	284.2^C^	21.3	280.0	256.0	310.0	257.8	310.6	
MNC	199.7^D^	13.6	199.0	184.5	219.0	182.8	216.6	

∗: Significant at P ≤ 0.05, Means with different superscript letters within the same vertical column are significantly different from each other.

**Figure 4:**
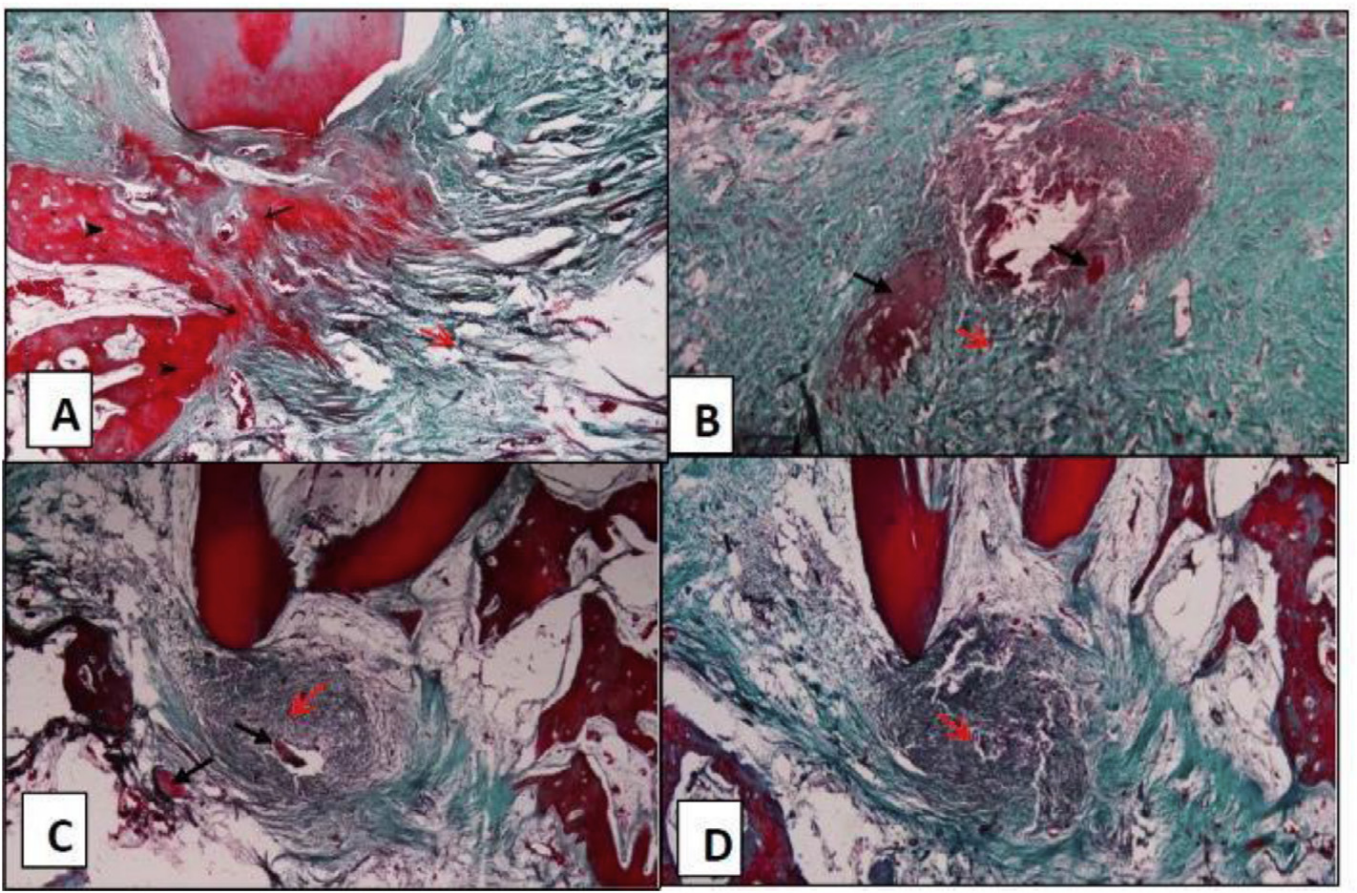
Light microscopic photo with Goldner Trichrome stain: (A)MNC: blue green stain (red arrows) represents newly formed collagen and granulation tissue while the older mature and bone stained red (black arrows), (B) Biogen: localized area of mature bone and collagen (black arrow) surrounded by totally immature collagen, (C) Collacote: same as B, (D) Control: totally newly formed collagen. (X40). (black arrows: newly formed bone, red arrows: granulation tissue).

The mean and standard deviation values are presented in Table 4. There was a statistically significant difference between values measured in different groups (P < 0.001). The highest value was found in MNC group (36.3 ± 4.4), followed by Biogen (24.9 ± 3.0), then Collacote (13.3 ± 1.4) while the lowest value was found in the control group (7.0 ± 1.6). Pairwise comparisons showed all groups to have a significantly different values from each other (P < 0.001) (Figure 4).

**Table 4:** The means, SD values of percentages of new bone in all groups.

Group	Mean	SD	Median	Minimum	Maximum	95% CI		*P*-value
						Lower bound	Upper bound	
Control	7.0^D^	1.6	7.0	5.0	9.0	5.0	9.0	**<0.001∗**
Collacote	13.3^C^	1.4	12.9	11.6	14.8	11.6	15.0	
Biogen	24.9^B^	3.0	24.0	22.0	28.6	21.2	28.6	
MNC	36.3^A^	4.4	36.0	30.0	41.0	30.9	41.8	

∗: Significant at P ≤ 0.05, Means with different superscript letters within the same vertical column are significantly different from each other.

## 4 Discussion

To overcome the fact that osseous regeneration of bone cavity will not occur, and therefore the defect will heal by fibrous connective tissue repair as stated by Andreason & Rud [22], many trials had been made. In 2002, Young *et al.* [23] stated that regenerating new tissue from isolated cells loaded on biocompatible scaffolds in the presence of growth factors is a promising method to enhance and accelerate regeneration. Since that, a lot of attempts addressed the cell therapy approach.

Cell therapy refers to cellular material with biological activities that cause the desired effect either in vitro or in vivo [10]. Cell therapy using BMMNCs is being used with promising results in preclinical and clinical approaches to treat bone defects [13, 24, 25]^.^

In this work, we used BMMNCs combined with the CollaCote collagen scaffold to evaluate their effect on the healing of bone cavities in-vivo. This combination provides an osteogenic, osteoconductive, and osteoinductive system.

The size of the bone cavities was standardized to be 15 mm × 10 mm x 10 mm as it was stated by Wang *et al.* [26] that cavities over 7 mm are complicated and cannot be effectively managed.

BM cells harvesting and implantation procedures are considered as simple, safe, and feasible methods [27]. Autologous bone marrow was simply obtained by aspiration from the iliac crest [14]. This aspiration contains undifferentiated mononuclear cells and multinuclear cells. The mononuclear cells can be either hematopoietic or mesenchymal, which may produce muscle cells, hepatocytes, adipose tissue and chondrocytes [28, 29]^‏^ In respect to the isolation of the different fractions of bone marrow cells, mononuclear cells are obtained with a simpler process than the mesenchymal cells, because they need only to pass through centrifugation [27, 30] while the mesenchymal cells need to pass through the same process followed by cell culture and expansion presenting much higher costs and a greater risk of infection and contamination [31, 32]. Moreover, the idea of probable collaboration between mononuclear cells and other BM cells in tissue repair and the existence of non-adherent osteogenic cells in the BM gave the use of BMMNCs an upper hand [33]. Furthermore, Granero-Molto *et al.* [34] stated that at the injury site, mesenchymal stem cells could help in repair in two ways; first by differentiating into tissue cells in order to restore lost morphology and function and second by secreting a wide spectrum of bioactive factors.

In this study we used CollaCote as a scaffold. Collagen scaffold is excellent for cell differentiation and proliferation as it allows easy placement of cells and growth factors [8]. Furthermore, it is totally replaced with natural tissues after degradation [35, 36]^.‏^

Bone grafts are considered as the gold standard for bone regeneration and being used with varying degrees of success. Biogen, which is an osteoconductive bone graft material, was applied in this study. It is widely used for maxillary sinus lift techniques and mandibular ridge augmentation with great success [37, 38]^.^

Our comprehensive search of the literature failed to reclaim any matching research equivalent with ours in terms of design and bone defects; nevertheless, other related studies are considered. The histological results of the present study revealed that there was statistically significant difference among the groups, when comparing the inflammatory cells infiltrate. The MNC group displayed less inflammatory cells infiltrates whereas the control group displayed more. This can be explained by the role that MNCs may play in regulating immune responses as stated by Stagg J [39]^.^ Also, Leal M *et al.* [40] found that these cells modulated the production of critical inflammatory mediators and decrease the inflammatory cells infiltration.

In the course of this investigation, bone regeneration in periapical defects was affected by the materials applied within the bony defect. A smaller bone defect was left, and more bone was formed when BMMNCs seeded on CollaCote were applied with less inflammatory cell count and better healing. Such findings agree with Becker S et al [41] and other researches that had proven that the autologous bone marrow in combination with the different scaffold was effective in promoting bone formation in various animal models such as dogs [42], rabbits [43] and mice [44]. Also, our findings are in line with Zou D et al. [45] who investigated the effect of using ordinary and genetically enhanced bone marrow mesenchymal stem cells on bone regeneration in critical-sized rat calvarial defects and showed dramatical improvement in bone volume, bone mineral density, blood vessel number, and blood vessel area compared to the control group.

On another hand, Henkel et al. [46] showed different results after grafting minipig mandibular defects with a bioactive matrix (60% hydroxyapatite and 40% β-tricalcium phosphate) alone or mixed with mesenchymal stem cells. They concluded that the addition of mesenchymal stem cells did not improve bone formation after an implantation period of 5 weeks. But this can be accredited to the difference in culturing method as the authors revealed that the nutrition of the cultured osteoblasts seeded in the carrier material was insufficient for complete ossification to occur.

The addressed therapeutic approaches specifically, cell therapy, look to beat the constraints of conventional therapies. The one proposed in this work combined two simple and available components, autologous BMMNCs from iliac crest BM and collagen scaffold convey an improvement in the healing of bone cavity in vivo.

## 5 Conclusion

Cell therapy approach using autologous BMMNCs from iliac crest BM and collagen scaffold minimized the inflammatory response and increased the area and volume of newly formed bone and should be more addressed in future research.

## Declarations

### Author contribution statement

Ehab Hassanien: Conceived and designed the experiments.

Sara El-Gindy: Performed the experiments.

Kareim Mostafa Elbatouty: Analyzed and interpreted the data.

Maram Farouk Obeid: Analyzed and interpreted the data; Contributed reagents, materials, analysis tools or data; Wrote the paper.

Elham Elshaboury: Contributed reagents, materials, analysis tools or data.

### Funding statement

This research did not receive any specific grant from funding agencies in the public, commercial, or not-for-profit sectors.

### Data availability statement

Data included in article/supplementary material/referenced in article.

### Declaration of interests statement

The authors declare no conflict of interest.

### Additional information

No additional information is available for this paper.
